# Handgrip strength and risk of malnutrition are associated with an increased risk of hospitalizations in inflammatory bowel disease patients

**DOI:** 10.1177/17562848231194395

**Published:** 2023-08-31

**Authors:** Katherine Bedard, Lorian Taylor, Naheed Rajabali, Karen Kroeker, Brendan Halloran, Guanmin Meng, Maitreyi Raman, Puneeta Tandon, Juan G. Abraldes, Farhad Peerani

**Affiliations:** Department of Medicine, Faculty of Graduate Studies and Research, University of Alberta, Edmonton, AB, Canada; Department of Medicine, University of Calgary, Calgary, AB, Canada; Division of Geriatric Medicine, University of Alberta, Edmonton, AB, Canada; Division of Gastroenterology, University of Alberta, Edmonton, AB, Canada; Division of Gastroenterology, University of Alberta, Edmonton, AB, Canada; Division of Gastroenterology, University of Alberta, Edmonton, AB, Canada; Department of Medicine, University of Calgary, Calgary, AB, Canada; Department of Community Health Sciences, University of Calgary, Calgary, AB, Canada; Division of Gastroenterology, University of Alberta, Edmonton, AB, Canada; Division of Gastroenterology, University of Alberta, Edmonton, AB, Canada; Division of Gastroenterology, University of Alberta, 1-59 Zeidler Ledcor Centre, 8540 – 112 Street NW, Edmonton AB T6G 2P8, Canada

**Keywords:** dynapenia, frailty, hospitalizations, inflammatory bowel disease, malnutrition

## Abstract

**Background:**

In patients with inflammatory bowel disease (IBD), frailty is independently associated with mortality and morbidity.

**Objectives:**

This study aimed to extend this work to determine the association between the clinical frailty scale (CFS), handgrip strength (HGS), and malnutrition with IBD-related hospitalizations and surgeries.

**Design:**

IBD patients ⩾18 years of age were prospectively enrolled from two ambulatory care clinics in Alberta, Canada.

**Methods:**

Frailty was defined as a CFS score ⩾4, dynapenia as HGS < 16 kg for females and <27 kg for males, malnutrition using the subjective global assessment (SGA), and the risk of malnutrition using either the abridged patient-generated SGA (abPG-SGA), or the Saskatchewan Inflammatory Bowel Disease Nutrition Risk Tool (SaskIBD-NRT). Logarithm relative hazard graphs and multivariable logistic regression models adjusting for relevant confounders were constructed.

**Results:**

One hundred sixty-one patients (35% ulcerative colitis, 65% Crohn’s disease) with a mean age of 42.2 (±15.9) years were followed over a mean period of 43.9 (±10.1) months. Twenty-seven patients were hospitalized, and 13 patients underwent IBD-related surgeries following baseline. While the CFS (aHR 1.34; *p* = 0.61) and SGA (aHR 0.81; *p* = 0.69) did not independently predict IBD-related hospitalizations, decreased HGS (aHR 3.96; *p* = 0.03), increased abPG-SGA score (aHR 1.07; *p* = 0.03) and a SaskIBD-NRT ⩾ 5 (aHR 4.49; *p* = 0.02) did. No variable was independently associated with IBD-related surgeries.

**Conclusion:**

HGS, the abPG-SGA, and the SaskIBD-NRT were independently associated with an increased risk of IBD-related hospitalizations. Future studies should aim to validate other frailty assessments in the IBD population in order to better tailor care for all IBD patients.

## Introduction

Inflammatory bowel disease (IBD), which includes both ulcerative colitis (UC) and Crohn’s disease (CD), is a chronic immune-mediated condition characterized by gastrointestinal inflammation that follows a relapsing and remitting course.^1^ While genetic predispositions and environmental factors both contribute to the development of IBD, the composition of the gut microbiome and the immune response also play a role in disease pathogenesis.^1–4^ The majority of cases are diagnosed in individuals 20–30 years of age; however, 10–15% of diagnoses occur in those who are at least 60 years of age.^5–7^ Although advanced age in patients with IBD has been reported to be associated with adverse clinical outcomes, such as inpatient morbidity, reduced efficacy of anti-tumor necrosis factor (anti-TNF) medications, and high healthcare resource utilization,^8–11^ chronological age may not always accurately reflect ‘biological age’, or the extent of physiological reserve an individual possesses to endure stressors.^12^

The effect of frailty on various chronic disease states has been well established;^13–15^ however, it is an emerging field within IBD. Having no gold standard definition, frailty is a concept that is often explained by a multidimensional state or syndrome including facets such as functional impairment, sarcopenia, and malnutrition.^16–21^ A recent systematic review demonstrated that frailty predicts hospitalizations and mortality-related outcomes in patients with IBD.^22^ As opposed to most frailty indices, which have been developed and validated in elderly patients, the Clinical Frailty Scale (CFS)^20^ is a judgment-based frailty screening tool that incorporates comorbidities, cognition, and function and may be more practical for IBD practitioners to use. While sarcopenia and malnutrition overlap with frailty, they have also been associated with the need for IBD surgery, increased post-operative complications and hospitalizations.^23,24^

To date, population-based health administrative data is utilized in most studies exploring the relationship between the multifaceted components of frailty and adverse IBD outcomes.^22^ The use of data in this form is subject to misclassification bias and fails to adequately account for disease severity. Therefore, there is a need in the IBD population to design prospective studies to provide a more robust association between frailty and its determinants with adverse clinical outcomes. Although reported rates of malnutrition in the IBD population vary based on the assessment tool used, it is universally accepted that those with IBD are subject to an increased risk of malnutrition.^25–29^ While the Subjective Global Assessment (SGA) is considered the current gold-standard for the diagnosis of malnutrition, self-screening tools such as the abridged patient-generated Subjective Global Assessment (abPG-SGA) and the Saskatchewan Inflammatory Bowel Disease Nutrition Risk Tool (SaskIBD-NRT) should be considered in order to improve the detection of malnutrition or malnutrition risk.^24,30^ The purpose of this study was to determine if frailty as defined by the CFS^20^ or determinants of frailty, as defined by handgrip strength (HGS),^31^ the SGA,^32^ the abPG-SGA,^33^ and the SaskIBD-NRT^34^ are independently associated with IBD-related hospitalizations or surgeries.

## Methods

### Study population selection

Patients with a confirmed diagnosis of either UC or CD followed at one of two IBD specialty ambulatory care clinics in Alberta, Canada were prospectively enrolled between May 2017 and March 2018. Consecutive patients were enrolled and followed until their last bloodwork, endoscopy, or clinic visit. Patients ⩾18 years of age with an IBD disease duration ⩾3 months at the time of enrollment were included in this study irrespective of clinical disease activity. While patients were not excluded from the study for hand anatomic or functional deformities, this would have been exclusionary for performance of the HGS measurement. Exclusion criteria for the study were as follows: (1) presence of a major medical comorbidity [chronic renal failure, chronic pulmonary disease, and congestive heart failure (ejection fraction <40%), or cirrhosis], (2) previous colectomy, (3) current pregnancy, or (4) inability to provide informed consent in English.

### Assessment of frailty and frailty determinants

The primary variable of interest was the CFS. Secondary variables of interest included HGS, the SGA, the abPG-SGA, and the SaskIBD-NRT. The CFS and HGS assessments were completed at baseline by trained research assistants, where the instruction to appropriately administer the CFS involved standardized verbal and written training. The ability of the trained assistants to accurately administer the CFS scores was confirmed through the direct monitoring of their performance.^35^ SGA groupings were determined by a registered dietician, and the abPG-SGA scores and SaskIBD-NRT groupings were self-reports completed by the patients. The CFS is a frailty assessment score based on clinical judgment incorporating comorbidities, disabilities, as well as cognitive and physical ailments ranging from one to nine (very fit to terminally ill).^20^ Patients with a CFS score ⩾4 were categorized as frail, which also included pre-frail patients.^20,36,37^ HGS measurements were taken to quantify the degree of decreased muscle strength or dynapenia, where the Jamar^®^ Hydraulic Hand Dynamometer was used to measure HGS in the dominant hand of each participant. Dynapenia was determined using the cut-offs of <16 kg and <27 kg, for female and male patients respectively.^38^ Malnutrition information was collected using the SGA, while malnutrition risk data were collected using the abPG-SGA and the SaskIBD-NRT. Those deemed as Moderate (B) or Severe (C) on the SGA were considered to be malnourished, whereas abPG-SGA and SaskIBD-NRT scores are from 0 to 35 and 0 to 9 respectively, where the cut-off designating risk for malnutrition was ⩾6 for the abPG-SGA and ⩾5 (high risk) for the SaskIBD-NRT.^33,34,39^

### Baseline characteristics

Baseline characteristics were obtained including age, sex, disease duration, follow-up length, previous IBD-related hospitalizations/surgeries, IBD type, Montreal classification,^40^ clinical disease activity, smoking status, IBD medication history, and body mass index (BMI). Laboratory assessments collected ±4 weeks from baseline included albumin, hemoglobin, and c-reactive protein (CRP).

### Outcomes

The primary outcome of interest for this study was IBD-related hospitalizations, and the secondary outcome was IBD-related surgeries. The following reasons for hospitalizations were included: disease flares requiring medical management, IBD-related surgeries, and infections. Similarly, the following reasons for IBD-related surgical procedures were included: disease activity, colorectal dysplasia, and colorectal cancer. The specific type of IBD-related surgery performed was captured including colectomy, small bowel resection/stricturoplasty, ileocecal resection, segmental colonic resection, and post-operative complications arising from a previous IBD-related surgery.

### Statistical analysis

To assess the impact of the exposures on the outcome, we used univariable and multivariable logistic regression and Cox models. Exposures were adjusted for the following potential confounders: age, sex, disease phenotype, disease activity [clinical remission determined by partial Mayo (pMayo)^41^ for UC or Harvey Bradshaw Index (HBI) scores^42^ for CD], exposure to biologics, exposure to steroids, previous IBD-related surgeries, and presence of comorbidities [determined using the Charlson *et al.* Comorbidity Index (CCI)^43^; categorized into no comorbidities and ⩾1 comorbidity]. Differences between baseline characteristics were determined using independent sample two-sided *t*-tests or Pearson’s chi-squared tests. A bivariate correlation test with a two-tailed test of significance was completed to analyze the possible correlation between frailty, sarcopenia, and malnutrition assessments, where Spearman correlation coefficients were used to indicate the extent of the correlations. To understand the relations between the different exposures, we used hierarchical cluster analysis based on Spearman correlations. Statistical analyses were performed using SPSS statistical software (v28) and R using the *rms* package.^44^

## Results

### Baseline characteristics

One hundred sixty-one patients with a mean age of 42.2 (±15.9) years were followed over a mean period of 43.9 (±10.1) months (Table 1). Most patients at baseline were on biologics (72.7%) in clinical remission [65% CD with a mean HBI score of 3.7 (±3.9) and 35% UC with a mean pMayo score of 1.3 (±1.8)] with a normal CRP <8.0 mg/L (79.2%). Notable differences between patients with UC and CD included the mean age [38.4 years (UC); 44.2 years (CD), *p* = 0.03], disease duration [120.1 months (UC); 169.8 (CD), *p* = 0.03], immunomodulator use [35.7% (UC); 48.6% (CD), *p* = 0.01], and current exposure to biologics [60.7% (UC); 79.0% (CD), *p* = 0.04]. Baseline characteristics of frailty, dynapenia, malnutrition, and malnutrition risk were similar between those with UC and CD (Supplemental Table 1).

**Table 1.** Baseline characteristics of patients.

Variable	Total patients (*n* = 161)	Ulcerative colitis patients (*n* = 56)	Crohn’s disease patients (*n* = 105)	*p*-Value*
Age				
Mean age, years (SD)	42.2 (15.9)	38.4 (15.8)	44.2 (15.7)	**0.03**
<60 years	137 (85.1%)	50 (89.3%)	87 (82.3%)	
⩾60 years	24 (14.9%)	6 (10.7%)	18 (17.1%)	
Sex				
Male	79 (49.1%)	27 (48.2%)	52 (49.5%)	0.88
Female	82 (50.9%)	29 (51.8%)	53 (50.5%)	
Disease duration				
Mean duration, months (SD)	152.5 (137.7)	120.1 (97.8)	169.8 (152.5)	**0.03**
Follow-up length				
Mean length, months (SD)	43.9 (10.1)	42.1 (9.8)	44.9 (10.2)	0.10
Disease activity				
pMayo mean score (SD)	–	1.3 (1.8)	–	–
HBI mean score (SD)	–	–	3.7 (3.9)	–
Montreal classification				
UC (*n* = 56)				–
E1	–	1 (1.8%)	–	
E2	–	15 (26.8%)	–	
E3	–	40 (71.4%)	–	
CD (*n* = 105)				
Age at diagnosis				–
⩽16 years	–	–	22 (21.0%)	
17–40 years	–	–	65 (61.9%)	
>40 years	–	–	18 (17.1%)	
Disease location				–
Terminal ileum	–	–	26 (24.8%)	
Colonic	–	–	21 (20.0%)	
Ileocolonic	–	–	58 (55.2%)	
Upper GI involvement				–
Yes	–	–	14 (13.3%)	
No	–	–	91 (86.7%)	
Disease behavior				–
Inflammatory	–	–	48 (45.7%)	
Stricturing	–	–	32 (30.5%)	
Penetrating	–	–	25 (23.8%)	
Perianal fistula(e)				–
Present	–	–	23 (21.9%)	
Absent	–	–	82 (78.1%)	
Previous IBD-related hospitalizations				
0, in previous 12 months	133 (82.6%)	47 (83.9%)	86 (81.9%)	0.89
⩾ 1, in previous 12 months	28 (17.4%)	9 (16.1%)	19 (18.1%)	
Previous IBD-related surgeries				
None	121 (75.5%)	56 (100.0%)^†^	65 (61.9%)	<**0.01**
Small bowel resection/stricturoplasty	20 (12.3%)	0 (0.0%)	20 (19.0%)	
Ileocecal resection	15 (9.2%)	0 (0.0%)	15 (14.3%)	
Segmental colonic resection	5 (3.1%)	0 (0.0%)	5 (4.8%)	
Laboratory tests				
Albumin (*n* = 68)				
Mean albumin, g/L (SD)	42.1 (3.8)	43.2 (3.1)	41.6 (4.0)	0.08
Hemoglobin (*n* = 122)				
Mean hemoglobin, g/L (SD)	138.4 (16.6)	137.8 (21.0)	138.6 (14.5)	0.95
C-reactive protein (*n* = 120)				
Mean c-reactive protein, mg/L (SD)	4.7 (7.5)	4.3 (6.2)	4.9 (7.7)	0.69
BMI				
Mean BMI, kg/m^2^ (SD)	26.7 (5.6)	26.5 (6.2)	26.8 (5.3)	0.70
Non-obese (BMI < 30 kg/m^2^)	129 (80.1%)	44 (78.6%)	85 (81.0%)	
Obese (BMI ⩾ 30 kg/m^2^)	32 (19.9%)	12 (21.4%)	20 (19.0%)	
Smoking status				
Non-smoker	85 (52.8%)	33 (58.9%)	52 (49.5%)	0.07
Previous smoker	27 (16.8%)	4 (7.1%)	23 (21.9%)	
Current smoker	49 (30.4%)	19 (33.9%)	30 (28.6%)	
Biologic medication				
None	38 (23.6%)	19 (33.9%)	19 (18.1%)	**0.04**
Previous	6 (3.7%)	3 (5.4%)	3 (2.9%)	
Current (*n* = 117)	117 (72.7%)	34 (60.7%)	83 (79.0%)	
Anti-TNF	114 (97.4%)	34 (100.0%)	80 (96.4%)	
Vedolizumab	1 (0.9%)	0 (0.0%)	1 (1.2%)	
Ustekinumab	2 (1.7%)	0 (0.0%)	2 (2.4%)	
5-ASA medication				
None	59 (36.6%)	7 (12.5%)	52 (49.5%)	<**0.01**
Previous	61 (37.9%)	21 (37.5%)	40 (38.1%)	
Current	41 (25.5%)	28 (50.0%)	13 (12.4%)	
Steroids				
None	44 (27.3%)	11 (19.6%)	33 (31.4%)	0.10
Previous	96 (59.6%)	34 (60.7%)	62 (59.0%)	
Current	21 (13.1%)	11 (19.6%)	10 (9.5%)	
Immunomodulators				
None	38 (23.6%)	21 (37.5%)	17 (16.2%)	**0.01**
Previous	52 (32.3%)	15 (26.8%)	37 (35.2%)	
Current	71 (44.1%)	20 (35.7%)	51 (48.6%)	

5-ASA, 5-aminosalicylic acid; anti-TNF, anti-tumor necrosis factor; BMI, body mass index; CD, Crohn’s disease; GI, gastrointestinal; HBI, Harvey-Bradshaw Index; IBD, inflammatory bowel disease; pMayo, partial mayo; SD, standard deviation; UC, ulcerative colitis.

* Significant differences between subgroups (UC and CD) at *p* < 0.05. Bold values indicate statistically significant differences between groups.

† UC patients who had previously undergone colectomy were excluded from the study.

### Effect of frailty and determinants of frailty on IBD-related hospitalizations

**Figure 1.**
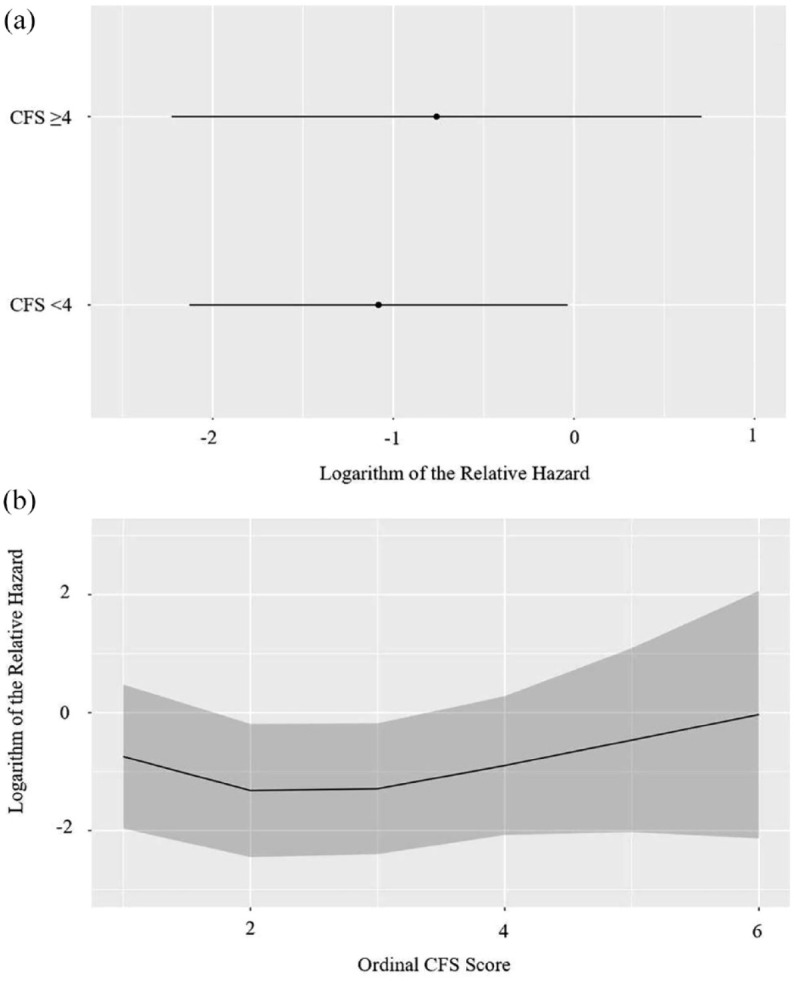
(a) Logarithm of the relative hazard graph demonstrating the risk of IBD-related hospitalizations between fit (CFS < 4) and frail (CFS ⩾ 4), (b) logarithm of the relative hazard graph demonstrating the risk of IBD-related hospitalizations as the ordinal CFS score increases. CFS, Clinical Frailty Scale.

**Figure 2.**
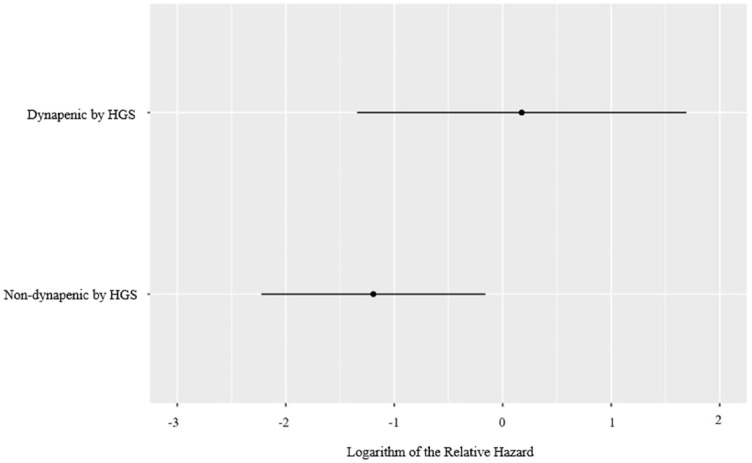
Logarithm of the relative hazard graph demonstrating the differing risk of IBD-related hospitalizations between non-dynapenic (HGS: ⩾16 kg for females, ⩾27 kg for males) and dynapenic (HGS: <16 kg for females, <27 kg for males) IBD patients. HGS, handgrip strength.

In total, 27 patients (16.8%) experienced at least 1 IBD-related hospitalization (disease flare, *n* = 14; IBD-related surgery, *n* = 10; infection, *n* = 3). Of these 27 patients, 5 were patients with UC and the remaining 22 were patients with CD. The CFS was not associated with IBD-related hospitalizations when used either ordinally (aHR 0.97; 95% CI, 0.66–1.43, *p* = 0.87) or categorically (aHR 1.34; 95% CI, 0.44–4.09, *p* = 0.61; Table 2). Logarithm relative hazard graphs illustrate the risk of IBD-related hospitalizations between the fit and frail patients (Figure 1(a)), as well as for the complete range of CFS scores (Figure 1(b)). In contrast to that of the CFS, decreased HGS was independently associated with an increased risk for IBD-related hospitalizations (aHR 3.96; 95% CI, 1.12–14.07, *p* = 0.03) (Table 2). Figure 2 illustrates the differences in the logarithm of the relative hazard of IBD-related hospitalizations for those classified by HGS as dynapenic versus those classified as non-dynapenic. Of note, neither BMI nor disease duration were associated with either the CFS or IBD-related hospitalizations (data not shown).

**Table 2.** Cox univariable HR and multivariable aHR for IBD-related hospitalizations and surgeries for all frailty, dynapenia, and malnutrition assessments.

Measurement	Outcome	Univariable HR (95% CI)	*p*-Value*	Multivariable aHR (95% CI)	*p*-Value*
***CFS*** Ordinal CFS score (1–9)	IBD-related hospitalizations	0.99 (0.69–1.41)	0.93	0.97 (0.66–1.43)	0.87
	IBD-related surgeries	1.04 (0.64–1.68)	0.89	0.95 (0.56–1.63)	0.86
CFS score ⩾4	IBD-related hospitalizations	1.61 (0.56–4.66)	0.38	1.34 (0.44–4.09)	0.61
	IBD-related surgeries	1.58 (0.35–7.11)	0.56	1.00 (0.20–5.00)	0.99
***HGS*** (females <16 kg, males <27 kg)	IBD-related hospitalizations	2.14 (0.74–6.20)	0.16	3.96 (1.12–14.07)	**0.03**
	IBD-related surgeries	2.04 (0.45–9.21)	0.35	3.50 (0.47–25.92)	0.22
***SGA***: Moderate malnutrition (B)	IBD-related hospitalizations	1.21 (0.46–3.19)	0.71	0.81 (0.28–2.35)	0.69
	IBD-related surgeries	0.93 (0.21–4.18)	0.92	0.43 (0.09–2.06)	0.29
***abPG-SGA*** Ordinal abPG-SGA score (0–35)	IBD-related hospitalizations	1.08 (1.03–1.14)	<**0.01**	1.07 (1.01–1.14)	**0.03**
	IBD-related surgeries	1.08 (1.01–1.15)	**0.03**	1.04 (0.96–1.13)	0.33
abPG-SGA Score ⩾6	IBD-related hospitalizations	2.30 (1.05–5.04)	**0.04**	1.99 (0.79–5.03)	0.15
	IBD-related surgeries	3.23 (1.08–9.61)	**0.04**	2.04 (0.59–7.06)	0.26
***SaskIBD-NRT*** Ordinal SaskIBD-NRT score (0–9)	IBD-related hospitalizations	1.37 (1.12–1.69)	<**0.01**	1.36 (1.05–1.77)	**0.02**
	IBD-related surgeries	1.48 (1.11–1.96)	<**0.01**	1.34 (0.92–1.94)	0.12
SaskIBD-NRT: high risk (Scores ⩾5)	IBD-related hospitalizations	4.02 (1.45–11.17)	**0.01**	4.49 (1.33–15.13)	**0.02**
	IBD-related surgeries	6.10 (1.46–25.52)	<**0.01**	4.28 (0.76–24.03)	0.10

abPG-SGA, abridged patient-generated Subjective Global Assessment; aHR, adjusted hazard ratio; CFS, Clinical Frailty Scale; CI, confidence interval; HGS, handgrip strength; HR, hazard ratio; IBD, inflammatory bowel disease; SaskIBD-NRT, Saskatchewan Inflammatory Bowel Disease Nutrition Risk Tool; SGA, Subjective Global Assessment.

* Significant HRs and aHRs at *p* < 0.05. Bold values indicate statistically significant HRs and aHRs.

**Figure 3.**
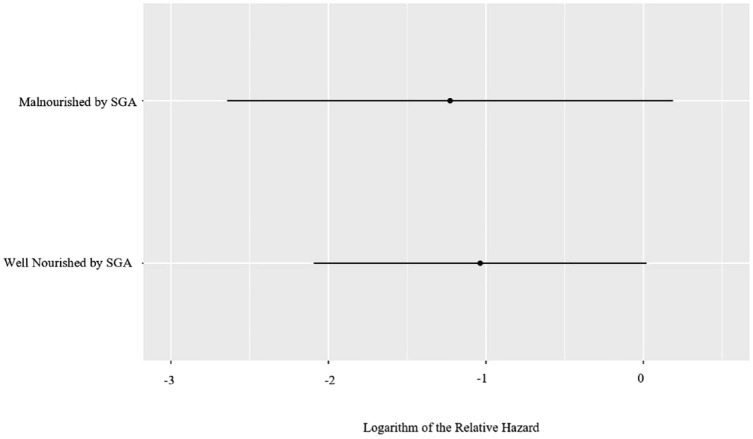
Logarithm of the relative hazard graph demonstrating the differing risk of IBD-related hospitalizations between well nourished (SGA-A) and malnourished (SGA-B) patients. SGA, Subjective Global Assessment.

**Figure 4.**
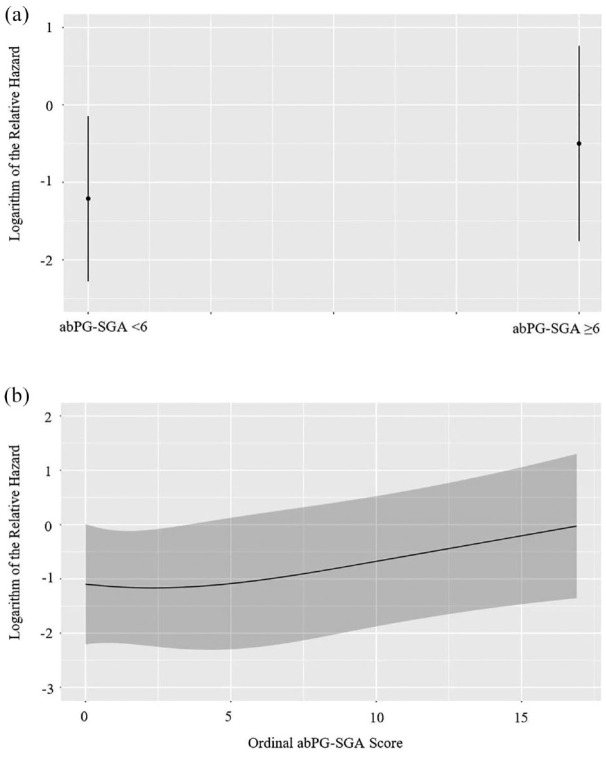
(a) Logarithm of the relative hazard graph demonstrating the risk of IBD-related hospitalizations between no risk of malnutrition (abPG-SGA < 6) and risk of malnutrition (abPG-SGA ⩾ 6), (b) logarithm of the relative hazard graph demonstrating the risk of IBD-related hospitalizations as ordinal abPG-SGA score increases. abPG-SGA, abridged patient-generated Subjective Global Assessment.

**Figure 5.**
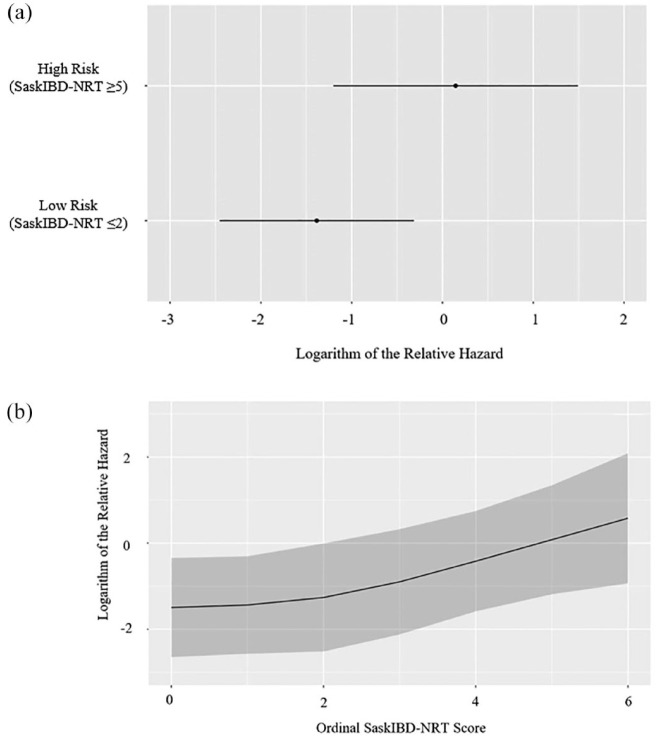
(a) Logarithm of the relative hazard graph demonstrating the risk of IBD-related hospitalizations between low risk of malnutrition (SaskIBD-NRT ⩽ 2) and high risk of malnutrition (SaskIBD-NRT ⩾ 5), (b) logarithm of the relative hazard graph demonstrating the risk of IBD-related hospitalizations as the ordinal SaskIBD-NRT score increases. SaskIBD-NRT, Saskatchewan Inflammatory Bowel Disease Nutrition Risk Tool.

With respect to malnutrition-related variables of interest, while the SGA was not associated with IBD-related hospitalizations [adjusted hazard ratio (aHR) 0.81; 95% CI, 0.28–2.35, *p* = 0.69], the abPG-SGA (aHR 1.07; 95% CI, 1.01–1.14, *p* = 0.03) and the SaskIBD-NRT (aHR 1.36; 95% CI, 1.05–1.77, *p* = 0.02) were (Table 2). Additionally, SaskIBD-NRT scores reflecting a high risk of malnutrition were significantly associated with IBD-related hospitalizations (aHR 4.49; 95% CI, 1.33–15.13, *p* = 0.02). Figures 3 to 5 illustrate the logarithm of the relative hazard of IBD-related hospitalizations with respect to the SGA, the abPG-SGA and the SaskIBD-NRT respectively.

### Effect of frailty and determinants of frailty on IBD-related surgeries

Thirteen patients (8.1%) underwent IBD-related surgeries, where two patients had UC and the remaining 11 were CD patients. As demonstrated by Table 2, no variable of interest was independently associated with IBD-related surgeries.

### Correlation between the CFS, HGS, SGA, abPG-SGA, SaskIBD-NRT, CCI, and age

**Figure 6.**
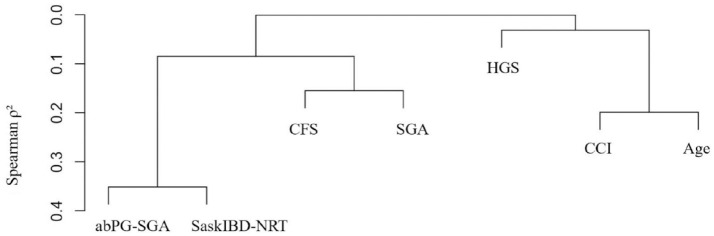
A dendrogram displaying the Spearman hierarchical clustering of the abPG-SGA, the SaskIBD-NRT, the CFS, the SGA, HGS, the CCI, and age. abPG-SGA, abridged patient-generated Subjective Global Assessment; CCI, Charlson Comorbidity Index; CFS, Clinical Frailty Scale; HGS, handgrip strength; SaskIBD-NRT, Saskatchewan Inflammatory Bowel Disease Nutrition Risk Tool; SGA, Subjective Global Assessment.

When all variables of interest were compared to each other, the abPG-SGA and SaskIBD-NRT had the highest Spearman correlation coefficient at 0.59 (*p* < 0.01) (Figure 6). The Spearman correlation coefficients between each of the remaining variables of interest were found to be low to moderate, with the exception of HGS with both the abPG-SGA (0.08, *p* = 0.34) and the SaskIBD-NRT (0.09, *p* = 0.24). All assessments and screening tools of interest in this study returned either non-significant *p*-values or negligible Spearman correlation coefficients when compared to chronological age. This contrasts with the low-moderate positive Spearman correlation coefficient of 0.45 (*p* < 0.01) present between the CCI and age.

## Discussion

In this prospective study, we observed that the CFS and SGA were not independently associated with IBD-related hospitalizations, whereas dynapenia as reflected by reduced HGS (aHR 3.96; *p* = 0.03), risk of malnutrition as represented by an increased abPG-SGA score (aHR 1.07; *p* = 0.03) and risk of malnutrition demonstrated through an increased SaskIBD-NRT score (aHR 1.36; *p* = 0.02) were (Table 2). In contrast, none of the variables of interest were associated with a significantly increased risk of IBD-related surgeries. To our knowledge, this is the first study with long-term follow-up exploring the independent effect of the CFS, HGS, abPG-SGA, and SaskIBD-NRT on IBD-related hospitalizations and surgeries.

Measuring frailty in the IBD literature has primarily been derived from frailty indices^22^ such as the Johns Hopkins Adjusted Clinical Groups Frailty Indicator^45^ and the Hospital Frailty Risk Score,^46^ which are scored from International Classification of Diseases, Ninth Revision (ICD-9) and International Classification of Diseases, Tenth Revision (ICD-10) codes. In our patient population, we used a bedside frailty tool known as the CFS,^20^ which is convenient, accurate, and considers clinical judgment.^20,47,48^ While demonstrated to be a useful frailty assessment, the CFS did not predict IBD-related hospitalizations or surgeries in our cohort. This could be related to the fact that only 10.4% of our patient population had CFS scores ⩾4 which would categorize them as being vulnerable to frailty, very mildly frail, or frail. This is not surprising as we excluded patients with severe pulmonary, renal, hepatic, or cardiac disease in the hopes of understanding the impact of frailty in this population related to IBD itself without additional major comorbidities. Moreover, as 72.7% of patients were on biologic therapy, the majority of whom were in clinical and biochemical remission, CFS scores may have been predictably low.^49^

Although the concepts of frailty, sarcopenia, and malnutrition are distinct, the multidimensional nature, complexity, and instability of frailty allow for sarcopenia and malnutrition to be considered as valid determinants of frailty.^38,50,51^ In our study, both ordinal and categorical scores for HGS and the various malnutrition assessment tools were reported, however the ordinal scores are preferred as they allow for a greater degree of reliability and precision in comparison to categorical scores.^52^ The variability and granularity in the abPG-SGA and SaskIBD-NRT risk of malnutrition scores likely explain why these tools demonstrated an increased risk of IBD-related hospitalizations with greater degrees of malnutrition and the SGA did not (Table 2). However, it should be recognized that over 80% of the patient population was considered well-nourished by the SGA, and none were deemed to be severely malnourished (Supplemental Table 1). While HGS alone was not associated with IBD-related surgeries in our study, sarcopenia as defined by HGS in conjunction with a physical performance assessment and appendicular skeletal muscle mass has previously been associated with higher rates of re-hospitalization (OR 5.50; *p* = 0.05) and surgery (OR 3.61; *p* = 0.02).^53^ It is evident that chronological age alone is not an all-encompassing prognostic marker for adverse IBD outcomes and that the concurrent use of other interdependent yet distinct facets of frailty may be useful (Figure 6).

This study has several notable strengths. First, this is the first prospective study that explored the effect of multiple bedside assessments of frailty, dynapenia, and malnutrition on adverse IBD outcomes. Second, this was a well-phenotyped cohort of patients followed over a mean length of almost 4 years. Third, while most previous studies exploring frailty have not been able to adequately control for the effect of disease severity,^22^ our prospective study provided granular detail regarding disease phenotype, previous hospitalizations, previous surgeries, and clinical as well as biochemical characteristics at baseline. Lastly, as demonstrated by Figure 6, the chosen frailty, dynapenia, and malnutrition-related constructs do not completely overlap, granting a multidimensional assessment, and unique prognostic abilities for each patient.

There are however limitations of this study that should be acknowledged. First, the low number of outcomes in this study may have impacted our ability to detect clinically significant differences in IBD-related surgeries. However, this was somewhat expected as most patients were in clinical remission. Second, the vast majority (85.1%) of our patient population was younger than 60 years of age. As the prevalence of frailty and its determinants increase with age, the frequency of patients deemed to be frail, dynapenic, or malnourished in our cohort was likely lower. Future studies should broaden the inclusion criteria to include patients with major medical comorbidities in order to increase the generalizability of our results. Third, the administration of CFS scores and HGS measurements by research assistants may not have been as precise compared to if they were administered by experienced clinicians. However, as noted by Shears *et al.*, the use of medical charts to determine CFS scores can be fulfilled by trained research staff.^54^ Though these assistants were trained as described above, no interrater reliability testing was completed for either assessment. Fourth, while the most accurate definition of sarcopenia includes low muscle quantity or quality along with an assessment of muscle function or strength,^38^ our study lacked a measurement of muscle quantity using such tools as dual-energy X-ray absorptiometry or computerized tomography scans. Fifth, though this study captured clinical disease activity and malnutrition parameters at baseline, the relationship between malnutrition and disease activity is complex and involves a cycle implicating the gut microbiome, inflammation, and immune modulation.^55,56^ This makes it challenging to identify true associations between either malnutrition or the risk of malnutrition and IBD-related hospitalizations or surgeries. Last, patients with a diagnosis of depression were not excluded, which has been reported to have a negative association with HGS.^57^

In conclusion, this is the first study with long-term follow-up to report significant independent associations between HGS, the abPG-SGA, and the SaskIBD-NRT with IBD-related hospitalizations in an outpatient population. These associations mirror findings from previous retrospective studies that utilized population-based administrative health data.^58–60^ Larger prospective cohorts of patients with IBD should be studied in the future, ideally focusing on an older patient population and allowing for the inclusion of patients with major medical comorbidities which may enrich the proportion of frail, sarcopenic, and malnourished patients. By identifying these types of patients, various programs such as surgical prehabilitation may be instituted to improve clinical IBD outcomes.^61–64^ While it is important to not only validate existing assessments and screening tools of frailty and its determinants in patients with IBD, the research community should also move forward to develop IBD-specific frailty tools to help clinicians better navigate care for the entire IBD population regardless of age.

## Supplemental Material

sj-docx-1-tag-10.1177_17562848231194395 – Supplemental material for Handgrip strength and risk of malnutrition are associated with an increased risk of hospitalizations in inflammatory bowel disease patients Supplemental material, sj-docx-1-tag-10.1177_17562848231194395 for Handgrip strength and risk of malnutrition are associated with an increased risk of hospitalizations in inflammatory bowel disease patients by Katherine Bedard, Lorian Taylor, Naheed Rajabali, Karen Kroeker, Brendan Halloran, Guanmin Meng, Maitreyi Raman, Puneeta Tandon, Juan G. Abraldes and Farhad Peerani in Therapeutic Advances in Gastroenterology

## Declarations

**Ethics approval and consent to participate:** The Health Research Ethics Boards at the University of Alberta (Pro00073470) and the University of Calgary (REB17-0890) both provided approval for this study. All participants provided informed consent prior to being enrolled in the study.

**Consent for publication:** Not applicable.

**Availability of data and materials:** The data that support the findings of this study are available from the corresponding author upon reasonable request.
