# Supplementary material for: Handgrip strength and risk of malnutrition are associated with an increased risk of hospitalizations in inflammatory bowel disease patients
Source: Ther Adv Gastroenterol. 2023 Aug 31;16:17562848231194395. doi: 10.1177/17562848231194395 (PMC10475242; doi:10.1177/17562848231194395)
Supplement: sj-docx-1-tag-10.1177_17562848231194395 – Supplemental material for Handgrip strength and risk of malnutrition are associated with an increased risk of hospitalizations in inflammatory bowel disease patients [file sj-docx-1-tag-10.1177_17562848231194395.docx]

| **Supplementary Table 1**. Baseline Frailty, Dynapenia and Malnutrition Characteristics of Patients | | | | |
| --- | --- | --- | --- | --- |
| **Characteristic** | **Total Patients (n=161)** | **Ulcerative Colitis Patients (n=56)** | **Crohn’s Disease Patients (n=105)** | **P-value*** |
| ***CFS***  Mean score (SD)  <4  ≥4 | 2.2 (1.1)  144 (89.4%)  17 (10.6%) | 2.1 (1.0)  53 (94.6%)  3 (5.4%) | 2.3 (1.1)  91 (86.7%)  14 (13.3%) | 0.50 |
| ***HGS***  Non-dynapenic  Dynapenic | 148 (91.9%)  13 (8.1%) | 49 (87.5%)  7 (12.5%) | 99 (94.3%)  6 (5.7%) | 0.13 |
| ***SGA***  Well (A)  Moderate (B)  Severe (C) | 134 (83.2%)  27 (16.8%)  0 (0.0%) | 44 (78.6%)  12 (21.4%)  0 (0.0%) | 90 (85.7%)  15 (14.3%)  0 (0.0%) | 0.25 |
| ***abPG-SGA***  Mean score (SD)  <6  ≥6 | 3.5 (5.5)  124 (77.0%)  37 (23.0%) | 2.4 (4.2)  45 (80.4%)  11 (19.6%) | 4.1 (6.1)  79 (75.2%)  26 (24.8%) | 0.05 |
| ***SaskIBD-NRT***  Mean score (SD)  Low Risk (≤2)  High Risk (≥5) | 1.6 (1.6)  119 (73.9%)  14 (8.7%) | 1.3 (1.6)  45 (80.4%)  4 (7.1%) | 1.8 (1.7)  74 (70.5%)  10 (9.5%) | 0.49 |

abPG-SGA, abridged patient-generated Subjective Global Assessment; CFS, Clinical Frailty Scale; HGS, handgrip strength; SaskIBD-NRT, Saskatchewan Inflammatory Bowel Disease Nutrition Risk Tool; SD, standard deviation; SGA, Subjective Global Assessment.

*Significant differences between subgroups (UC and CD) at P<0.05. Bold values indicate statistically significant differences between groups.
